# Injecting-related health harms and overuse of acidifiers among people who inject heroin and crack cocaine in London: a mixed-methods study

**DOI:** 10.1186/s12954-019-0330-6

**Published:** 2019-11-13

**Authors:** Magdalena Harris, Jenny Scott, Talen Wright, Rachel Brathwaite, Daniel Ciccarone, Vivian Hope

**Affiliations:** 1 Department of Public Health, Environments and Society, London School of Hygiene and Tropical Medicine, 15-17 Tavistock Place, London, WC1H 9SH UK; 2 Department of Pharmacy and Pharmacology, University of Bath, Claverton Down, Bath, BA2 7AY UK; 3 Social, Genetic and Developmental Psychiatry Centre, Institute of Psychiatry, Psychology and Neuroscience, King’s College London, London, UK; 4 School of Medicine, University of California, San Francisco, 513 Parnassus Ave, San Francisco, CA 94143-0410 USA; 5 Public Health Institute, Liverpool John Moores University, Tithebarn Street, Liverpool, L2 2QP UK

**Keywords:** Citric acid, Ascorbic acid, Vitamin C, People who inject drugs, Skin and soft tissue infections, Heroin, Vein damage, Harm reduction

## Abstract

**Background:**

Venous access is a priority for people who inject drugs (PWID). Damage and scarring of peripheral veins can exacerbate health harms, such as skin and soft tissue infections (SSTI), and promote transitions to femoral and subcutaneous injecting. Brown heroin available in Europe requires acidification for injection preparation. In this paper, we present mixed-methods data to explore our hypothesis of a link between overly acidic injection solutions, venous damage and SSTI risk.

**Methods:**

We present a structured survey (*n* = 455) and in-depth qualitative interview (*n* = 31) data generated with PWID in London for the Care & Prevent study. Participants provided life history data and detail on injecting environments and drug preparation practices, including the use of acidifiers. Bivariate and multivariate analyses were conducted using a logistic regression for binary outcomes to explore associations between outcomes and excessive acidifier use. Grounded theory principles informed inductive qualitative analysis. Mixed-methods triangulation was iterative with results comparison informing the direction and questions asked of further analyses.

**Results:**

Of the 455 participants, most (92%) injected heroin and/or crack cocaine, with 84% using citric as their primary acid for drug preparation. Overuse of acidifier was common: of the 418 who provided an estimate, 36% (*n* = 150) used more than ½ a sachet, with 30% (*n* = 127) using a whole sachet or more. We found associations between acidifier overuse, femoral injecting and DVT, but not SSTI. Qualitative accounts highlight the role of poor heroin quality, crack cocaine use, information and manufacturing constraints in acidifier overuse. Painful injections and damage to peripheral veins were common and often attributed to the use of citric acid.

**Conclusions:**

To reduce injecting-related injury and associated consequences, it is crucial to understand the interplay of environmental and practice-based risks underpinning venous damage among PWID. Overuse of acidifier is a modifiable risk factor. In the absence of structural supports such as safe injecting facilities or the prescribing of pharmaceutical diamorphine, there is an urgent need to revisit injecting paraphernalia design and distribution in order to alleviate health harms and distress among the most marginalised.

## Background

‘Care & Prevent’ is a five-phase mixed-methods study [1] that explores skin and soft tissue infection (SSTI) risk, sequelae, care and prevention with people who inject drugs (PWID) in London. In this paper, the first in a series on Care & Prevent findings, we explore associations between acidifier use and SSTI risk, unpack detailed accounts of injection preparation practice and discuss options for reducing acidifier related harm, using survey and qualitative data from phases 2 and 4, respectively. We focus on acidifier use because we hypothesise a link between overly acidic injection solutions, venous damage and SSTI risk.

Acid is necessary to prepare brown heroin (used in Europe) and crack cocaine for injection, as these base form drugs are poorly soluble in water. The addition of acid promotes conversion of heroin and/or crack into a soluble, injectable form [2]. The exact amount of acid needed to dissolve the psychoactive opiates in heroin varies depending on drug quality and weight as well as type of acidifier used. Laboratory experiments using heroin sourced through United Kingdom (UK) street markets illustrate that 27 mg of citric acid (‘two pinches’) or 67 mg of vitamin C (ascorbic acid) is sufficient to dissolve the 140 mg of diamorphine base present in a 250-mg heroin sample (selected as in upper weight range of £10 heroin samples) [2]. Both amounts, however, are smaller than the 100-mg citric acid and 300-mg vitamin C sachets^1^ provided for injection preparation in the UK. Promoted as ‘single use’, the 100-mg sachet weight is dictated by manufacturing process constraints, not because it is the amount required to prepare a typical single heroin injection [2, 3]. With the proviso that injections also including crack cocaine require additional acidifier, we posit that the use of more than half a sachet of citric acid or vitamin C is extraneous to the dissolution requirements of a typical £10 bag of street heroin for injection and is potentially causative of harm.

Prior to 2003, the provision of citric acid and vitamin C for injection preparation was in contravention of the UK law. PWID typically used household acids, such as lemon juice and vinegar, and purchased tubs of citric acid and vitamin C through pharmacies and home brew suppliers, where available. In 2000, retail access to citric acid and vitamin C declined markedly after the *Pharmaceutical Journal* published a letter from a pharmacist raising concerns about the legality of acidifier supply [4]. Agreements to protect against prosecution were sought and formalised in some localities [2, 3], with PWID increasingly using lemon and vinegar in others. The use of lemon juice in injection preparation can cause the fungal eye infection *Candida endophthalmitis*, with outbreaks documented among PWID in the 1980s and 1990s [5]. New reports of eyesight problems and blindness among PWID prompted the development and launch of single-use citric acid sachets in 2001 and vitamin C sachets in 2003 [6]. Amendments to section 9A of the Misuse of Drugs Act legalised their supply through medical and harm reduction providers in 2003 (citric) and 2005 (vitamin C).

Early evaluations of citric acid supply for injection in the UK showed that the availability of citric acid sachets attracted PWID into needle and syringe programs (NSPs) [3] and increased attendance frequency [7], indicating acceptability and widespread uptake. However, since this early interest—largely precipitated by the law change—there has been little research exploring the role of acidifier in injection preparation or its potential role in exacerbating venous damage and SSTI risk. Legalised provision of acidifier for injection in the UK only arose after concerted lobbying by harm reduction organisations and providers concerned about the health harms of household acid use. As a hard-won and necessary intervention, the potential of citric acid and vitamin C to produce, as well as avert, injecting-related harm was understandably overlooked.

A limited literature indicates associations between vein damage and acidifier use during injection preparation. Ciccarone and Harris [8] tested their hypothesis of a causal link between heroin solution acidity and venous sclerosis in an exploratory study with PWID in London and Philadelphia. They took still and video photographs of the heroin preparation process, asking each participant to provide a small sample to be tested for pH before being interviewed about their injecting practice. UK heroin solutions prepared with citric acid had a high acidity (average pH = 2.6), close to the acidity of wine vinegar, and almost ten times more acidic than solutions prepared with vitamin C (avg. pH = 3.4). Pharmaceutical heroin was the least acidic (pH =4.8); similar to injections prepared in Philadelphia (avg. pH = 4.7) where Columbian-sourced powder heroin (hydrochloride salt form) predominates. Salt form heroin, unlike the Afghanistan-sourced base heroin used in the UK, is highly soluble and does not require acidifier for injection preparation. London participants related painful injections and vein damage to citric acid use, with Philadelphia participants reporting minimal vein damage or injecting-related injury from heroin injecting. Three UK studies report PWID participants commonly using a whole sachet of citric acid for each heroin injection [3, 9, 10]. Qualitative studies illustrate uncertainty among PWID about how much acid it is appropriate to use for injection preparation and attributions of vein damage to citric acid use [8, 11].

Understanding the causes of venous sclerosis is fundamental to harm reduction initiatives for PWID. Damage and sclerosis of peripheral veins can lead to multiple injection attempts, increased blood in the environment and transitions to femoral and subcutaneous injecting [8, 12, 13]. Femoral vein injecting increases risk of venous insufficiency and deep vein thrombosis (DVT) [14, 15], which can lead to venous ulcers, tissue necrosis, amputation and potentially fatal pulmonary embolism. Subcutaneous injecting is associated with SSTI such as abscesses and cellulitis. SSTI are an increasing cause of acute and chronic morbidity among PWID [16–18]. Cross-sectional prevalence in the UK and US ranges from 10 to 36%, with lifetime prevalence as high as 70% [19–22]. National UK data from 2014 evidence 10% of PWID reporting hospital admission for SSTI in the preceding year [17, 23] with hospitalisations rising annually since 2012 [18]. Damaged veins not only precipitate health harms, such as hepatitis C, venous ulcers and SSTI, but are an acute source of suffering for PWID [13]. Interventions attending to immediate priorities of PWID, such as maintaining venous access, are crucial and likely to have more currency than those focused on long-term risks [13].

## Methods

In this paper, we present structured survey and in-depth qualitative interview data generated with PWID in London for the ongoing Care & Prevent study. Study methodology details are published [1]. Participants were eligible if aged 18 years and older and had a history of injecting psychoactive drugs. Recruitment took place through drug treatment services, homeless hostels and day centres across London. Interested participants learnt about the study through either service staff, recruitment flyers or an affiliated outreach team. Participants received a £10 voucher for answering survey questions and providing a urine sample to be tested for albuminuria (see [1] for details), and £20 for an interview. Four hundred and fifty-five PWID took part in the survey and 31 in qualitative interviews. Questionnaire and interview data were generated concurrently between October 2017 and March 2019. Analysis followed steps outlined for convergent design projects whereby each dataset is initially analysed separately using the appropriate qualitative and quantitative analytic methods, with results compared to inform the direction and questions asked of further analyses and data generation [24]. Final triangulation of mixed-methods data prioritised complementarity (findings greater than the sum of their parts), while also attentive to convergence and dissonance [25].

The questionnaire was prepared using the Open Data Kit (ODK) software and administered using the ODK Collect application on Android tablets [26] by trained researchers. Participants answered questions pertaining to their: socio-demographics; drug use history; injection preparation and administration practices (lifetime and previous 12 months); reuse and cleaning of injecting equipment (lifetime); experience of SSTIs and other health conditions; care-seeking and hospitalisation (lifetime). Using Stata version 15.1, the characteristics of study population and the prevalence risk factors were described using numbers and percentages for categorical variables, means (standard deviations) for normally distributed continuous variables and median (95% CI) for non-normally distributed continuous variables. Bivariate and multivariate analyses were conducted using a logistic regression for binary outcomes to explore association between outcomes and excessive acidifier use. A priori, we proposed the following outcomes could be associated with using excessive acid use: injection sites related to vein loss, e.g. groin injecting and non-antecubital fossa (inner elbow) injecting, attempts at vein injecting, SSTIs and sequelae of SSTIs, e.g. microalbuminuria. Where possible associations were found in the bivariate analyses (*p* < 0.10), these were further explored in multivariable analyses to adjust for the following possible confounders: age, gender, and injecting frequency per week.

Questionnaire participants were purposively sampled for invitation to the qualitative interview, with attention to capturing diversity in age, gender, injecting history and experience of SSTI. Interviews were of 60–120 min in duration, audio-recorded with consent and conducted in a private room at a recruiting service, a café or the participant’s home. Participants were invited to talk about their life history and their drug use trajectory. Detail was sought about injecting environments and drug preparation practices, including the use of acidifiers. Field-notes were generated after each interview, noting participant-interviewer dynamics, environmental context and interview content to follow-up on or consider in analysis. Interview audio-recordings were transcribed verbatim, cleaned to ascertain anonymity and entered into NVivo 12 for data management and analysis.

Qualitative analysis was informed by constructivist grounded theory methods [27] with data analysed as generated in order to inform the direction of subsequent interviews, coding, case selection, memo and theory generation. Initially, M. H open coded five transcripts line-by-line using process or gerund codes [27]. In consultation with team members, inductive open-codes were consolidated into focused codes. These formed the basis of a coding frame, comprising 13 ‘first-level’ codes or categories. The coding frame was entered into NVivo, and two researchers coded the same four interviews against the 13 categories before coding independently. The second-stage coding comprised inductive open coding of the data in each category to inform analytic interpretation and theme development. For example, the first-level category ‘Describing and accounting for acidifier use’ comprised 46 pages of data. Reanalysis demarcated the following second level codes: ‘accounting for overuse’, ‘attributing pain and damage’, ‘obtaining acidifiers and/or expressing preference’ and ‘taking care’. Consideration and comparison of the second-level code data then informed questions asked of quantitative analysis, with finding triangulation from each method generating the themes and analyses presented in this paper. In this way, analysis has been a detailed, iterative process, conducted in collaboration and with attention to consistency as well as movement toward theory generation and transferability.

### Ethical approval

Ethical approval for this study was granted by the London School of Hygiene and Tropical Medicine Observational Research Ethics Committee [12021], the London Bridge Research Ethics Committee and Health Research Authority [17/LO/0872]. All participants provided written consent after receiving study information and assurance of confidentiality.

## Results

### Participant demographics and injection practices

In total, 455 PWID completed the questionnaire across all study locations (see Tables 1 and 2 for participant characteristics). Participants were predominately male (75%, *n* = 341), of White ethnicity (74%, *n* = 336), and reflected the ageing population of PWID in the UK, with a mean age of 46 years. The majority were unstably housed with 32% (*n* = 146) currently (past 12 months) living in hostels, 13% (*n* = 61) street homeless and 3% (*n* = 15) in prison or staying with friends/family (6%, *n* = 28). Lifetime history of street homelessness was high, at 78% (*n* = 355). Participants were asked about lifetime experience of abscesses, cellulitis, venous ulcers and venous disease. The majority (65% *n* = 296) had experienced at least one of these conditions, of whom 46% (*n* = 137) reported hospitalisation. Two-thirds (62%, *n* = 284) reported injecting in the past 12 months, with a heroin and crack combination favoured by most (58%, *n* = 164). On average, participants had commenced injecting when 25 years old, 42% (*n* = 192) reported injecting their primary drug for 15 or more years. The majority (79%, *n* = 360) were currently on opiate substitution therapy, for a median duration of 10 years. Citric acid was the most commonly used acidifier during drug preparation (84%, *n* = 237). Participants were asked how much acidifier they would typically use to prepare a £10 bag of heroin, of the 418 who provided an estimate, excessive acid use was common with 36% (*n* = 150) reporting using more than ½ a sachet, of these 85% (*n* = 127) used a whole sachet or more.

**Table 1:** Injecting and treatment history: PWID London 2017–2019

Demographics and injecting history	*N* = 455
Demographics	*N* (%)
Age (years), mean (SD)	45.7 (9.0)
Gender	
Male	341 (75.0)
Female	114 (25.0)
Ethnicity	
White or White British	336 (73.9)
African, Caribbean or Black British	50 (11.0)
Asian or Asian British	12 (2.6)
Mixed ethnicity	27 (5.9)
Other	30 (6.6)
Place of residence, past 12 months	
Own house/flat	169 (37.1)
Shared house/flat	25 (5.5)
At parents, relatives or friends house	28 (6.2)
Hostel	146 (32.1)
Street homeless	61 (13.4)
Gaol/prison	15 (3.3)
Other	11 (2.4)
Ever homeless	
No	100 (22.0)
Yes	355 (78.0)
Injecting history	
Age of injecting initiation, mean (SD)	24.8 (8.7)
Duration injecting main drug	
One year or less	57 (12.5)
2–4 years	70 (15.4)
5–7 years	50 (11.0)
8–10 years	55 (12.1)
11–14 years	31 (6.8)
15+ years	192 (42.2)
Injected in the last 12 months	
Yes	284 (62.4)
No	171 (37.6)
Main drug injected in last 12 months	*N* = 284
Heroin and crack combined	164 (57.8)
Heroin only	97 (34.2)
Stimulants (amphetamine, methamphetamines, speed)	11 (3.9)
Crack only	5 (1.8)
Methadone	4 (1.4)
Cocaine	2 (0.7)
Methedrone	1 (0.4)
Main drug injected in the past, if had not injected in last 12 months	*n* = 171
Heroin only	102 (59.7)
Heroin and crack combined	61 (35.7)
Stimulants (amphetamine, methamphetamines, speed)	4 (2.3)
Crack (only)	2 (1.2)
Methadone	1 (0.6)
Steroids or other performance enhancing drugs	1 (0.6)
Treatment history	
Ever been on opiate substitution therapy (OST)	
Yes, currently	360 (79.1)
Yes, not now but in the past	62 (13.6)
No	33 (7.3)
Duration on opiate substitution therapy, if receiving OST currently	*n* = 360
Duration on OST (years), median (95% CI)	10 (7.10)

**Table 2:** Distribution of usual injection preparation and practices: PWID London 2017–2019

Injecting preparation, patterns and health outcomes	*N* = 455
	*n* (%)
Main acid used to dissolve drugs for injecting in the last 12 months	*n* = 284
Citric acid	237 (83.5)
Vitamin C	27 (9.5)
Lemon juice	4 (1.4)
Kettle descaler	1 (0.4)
No acid	15 (5.2)
Typical (usual) quantity of citric acid or vitamin C used to prepare a £10 bag of heroin	*n* = 418
More than a sachet	5 (1.2)
A whole sachet	122 (29.2)
¾ of a sachet	23 (5.5)
½ a sachet	136 (32.5)
¼ of a sachet	41 (9.8)
A pinch	91 (21.8)
Body site injected over lifetime	
Hands	333 (73.2)
Arms	439 (96.5)
Neck	169 (37.1)
Groin	189 (41.5)
Legs	275 (60.4)
Feet	251 (55.2)
Other	63 (13.9)
Frequency of injecting in a typical week	
Once a week	57 (12.5)
2–4 times a week	50 (11.0)
4–6 times a week	31 (6.8)
Once a day	44 (9.7)
2–3 times a day	164 (36.0)
4 or more times a day	109 (24.0)
Number of times usually insert the needle before successful vein injection	
Once (I always get it the first time)	202 (44.4)
Twice	82 (18.0)
3–4 times	58 (12.8)
4 or more times	108 (23.7)
I do not inject into a vein	5 (1.1)
SSTI history and hospitalisation	
No	159 (35.0)
Yes, but not hospitalised	159 (35.0)
Yes, and hospitalised	137 (30.0)
Ever diagnosed with DVT	*N* = 455
No	350 (76.9)
Yes	105 (23.1)
Ever diagnosed with blood poisoning	
No	372 (81.8)
Yes	83 (18.2)
Ever diagnosed with endocarditis	
No	433 (95.2)
Yes	22 (4.8)
Current albumin-creatinine ratio (past 12 months)	*N* = 316
Normal	254 (80.4)
Abnormal/high abnormal	62 (19.6)

Qualitative interview participants (*n* = 31) reflected the demographics of the larger sample. Most were men (71%, *n* = 22), of White ethnicity (81%, *n* = 25), and with a mean age of 43 years. The mean age at first injection was 23 years, with half (51%, *n* = 16) injecting their primary drug for 15 or more years. Of the 30 participants asked about acidifier use, most used citric acid (77% *n* = 23), with a similar proportion as the larger sample using more than half a sachet of acidifier (37%, *n* = 11). Of these, most reported using a whole sachet or more (82% *n* = 9).

### Injecting practices and health outcomes associated with acidifier over-use

Bivariate analyses found that those who reported excessive acidifier use (more than half a sachet of acidifier) had increased odds of injecting in their groin (OR 1.92 *p* = 0.002), being diagnosed with DVT (OR 1.82, *p* = 0.010), experiencing septicaemia (OR 1.59, *p* = 0.0.068), testing positive for micro/macroalbuminuria (indicative of prolonged SSTI or other source of inflammation) (OR 1.85, *p* = 0.041) and being diagnosed with endocarditis (OR 2.04 0 = 0.113), than those who used less (see Table 3). No association was found between acidifier overuse and SSTI history [OR 1.03, *p* = 0.912], ever injecting in any other body parts, legs (OR 1.15, *p* = 0.491), feet (OR 1.08, *p* = 0.699), hands (OR 0.87 (*p* = 0.484), and arms (OR 0.69, *p* = 0.591), and accessing a vein on the first attempt versus multiple attempts (OR 0.87, *p* = 0.484). After adjusting for possible confounders in step-wise multivariable analyses, excessive acidifier use was associated with increased odds of injecting in the groin [AOR 1.95 (95% CI 1.29, 2.95)], diagnosis of DVT [AOR 1.87 (95% CI 1.18, 2.97)], and micro/macroalbuminuria [AOR 1.85 (95% CI 1.02, 3.35)]. Diagnoses of blood poisoning and endocarditis did not reach traditional significance (*p* < 0.05) after adjustment.

**Table 3:** Unadjusted and adjusted odds ratios (OR) with 95% CI for the risk of groin injecting, micro/macroalbuminuria, diagnoses of DVT and septicaemia among high acidifier use

	Low acidifier^a^ (%)	High acidifier^b^ (%)	OR bivariate (95% CI)	*P* value	AOR multivariate^c^ (95% CI)	*P* value
Ever injected in groin						
No	168 (63%)	70 (47%)	1	–	1	–
Yes	100 (37%)	80 (53%)	1.92 (1.28, 2.88)	0.002*	1.95 (1.29, 2.95)	0.002*
Ever diagnosed with DVT						
No	213 (79%)	102 (68%)	1	–	1	–
Yes	55 (21%)	48 (32%)	1.82 (1.16, 2.87)	0.010*	1.87 (1.18, 2.97)	0.008*
Current Micro/macroalbuminuria^d^						
No	152 (64%)	85 (36%)	1	–	1	–
Yes	27 (49%)	28 (51%)	1.85 (1.03, 3.35)	0.041*	1.85 (1.02, 3.35)	0.042*
Ever diagnosed Septicaemia						
No	225 (84%)	115 (77%)	1	–	1	–
Yes	43 (16%)	35 (23%)	1.59 (0.97, 2.63)	0.068	1.60 (0.96, 2.67)	0.070
Ever diagnosed Endocarditis						
No	258 (96%)	139 (93%)	1	–	1	–
Yes	10 (4%)	11 (7%)	2.04 (0.85, 4.93)	0.113	2.27 (0.91, 5.66)	0.077

*OR* odds ratio*, AOR* adjusted odds ratio, *DVT* deep vein thrombosis

^a^Typically uses half or less of acid in drug preparation

^b^Typically uses more than half of acid in drug preparation

^c^Adjusted for age, gender and injecting frequency per week

^d^292/316 responses for acid use among those who provided urine samples

*Significant at *p* < 0.05

### Qualitative findings: accounting for (over)use of acidifier

We hypothesise that excessive acidifier use plays a causal role in venous irritation, damage and associated complications. It is therefore of interest to explore how modifiable this practice is. This requires an understanding of the role acidifier (over)use plays for participants, and the social relations and contexts in which injection preparation sits. The following section explores excessive acidifier use (as either participant or researcher-identified) through participant accounts of injecting preparation expertise, constraint and convention, and pain and practice modification. While not mutually exclusive, these themes indicate three distinct and situated rationalities of acidifier use with implications for intervention development and implementation.

#### Expertise: crack and cutting agents

Some participants adopted an educative role in the interview encounter, drawing on personal experience to evidence injecting preperation expertise and explain additional acidifier need. This was primarily in relation to the demands of preparing injections with poor quality heroin or crack cocaine (‘white’). As Troy says:

When you’re doing snowballs [heroin and crack] you have to have a bit more citric … like the white won’t dissolve if you don’t have enough citric in it.

Mason also educates on the need for acidifier dependant on drug type and heroin form:

Right, see when you use crack you need to put citric in to dissolve it, see when you use coke it just dissolves … the brown heroin is the same as the white heroin … It’s just cheaper to not to [process] it, so they just leave it brown, it’s cheaper to make and all you do is put citric in it.

Here, heroin refinement and purity are closely tied to the need for acidifier. These accounts often link to early injecting experiences. Marc recalls when he started injecting in the 1970’s: “It was pretty strong stuff. I mean I didn’t use any substance to dissolve it. We just heated it up on the spoon, with water, you know”*.* Although he later refers to this heroin as ‘brown’ indicating Afghani heroin (requiring acidification), other narratives indicate the presence of different heroin forms (such as salt form, non-smokable, heroin) in the ‘early days’. Ryan explains why he uses more acidifier now: “The gear [heroin] was better, like when I started using gear it wasn’t smoked, you know what I mean, it wouldn’t burn on the foil, yeah proper, proper gear.”

Expert accounts convey conviction and a strong rationale for additional acidifier use. For many, however, this rationale was undercut by a tension – between knowledge in theory and preference in practice. Mason speaks of the need for more acidifier when preparing heroin of poor quality, while simultaneously reflecting that, in effect, all this acted to do was break down less soluble cutting agents. His account deflects the problematics of this practice by framing it in terms of what ‘most people’ do, while briefly acknowledging that it is also part of his routine:

Most people think a sachet is for a bag … but see what the problem is, see all the crap that goes into smack, me and a lot of people, see when you cook up you’re trying to get that to dissolve because you don’t know what it is, yeah. You put more fucking citric in it and in reality, it’s not going to dissolve, but it could be anything, do you know what I mean, so that’s how people use loads of citric.

Dev also acknowledges that excess acidifier is used to break down cutting agents, moving from the third to first person to express this as a shared practice: “Yeah, you’re a user know what I mean, it’s going to be in your head, because you don’t want to waste no little bit, know what I mean, I want every little bit.” Logan would use up to two sachets of citric to prepare an injection, saying: “Just see how it breaks down, put like a sachet in, if it doesn’t break down you add more”. He was aware that this was ‘probably’ too much: “but when you see it [‘crap’] in the spoon, you just think, bugger it”*.* Some participants, such as Matt, spoke with equal certainty of the need to use more acidifier for poor quality heroin, but without necessarily the awareness that this is problematic:

Sometimes you [need more] depending on the gear. Because I know how much citric I’ve used, and I’m used to how much I put on, and sometimes like you cook it up and you can tell that you need to add a little bit more to cook it up further and just depends what they cut the gear with I guess.

Dean, who initially draws on his expertise to explain how risk is identified (foil running red) and mitigated against (avoiding citric), further illustrates the tension between expert knowledge and routinised practice:

My brother’s a smoker, so I can see on his foil that it’s running red, so I know it’s got shit in it already and like if I ain’t got Vit C and I have to use lemon, all lemon will cook up is the gear, it won’t wash all the other shit they put with it. So you can see it all in the bottom of the cup, like all the crap. Citric generally washes everything up, even the shit, so you don’t really know. When you use lemon juice or vinegar it just washes the gear up and leaves all the shit in the bottom of the cup.

While the above account indicates care taken to avoid injecting impurities, Dean’s preference in practice undermines this impression:

I prefer citric, to be honest, but if I was in my logical mind I’d use Vit C rather, because that doesn’t wash all the impurities up, that just washes all the actual gear and then all the shit that’s in it, it leaves in the bottom of the cup as well with Vit C.

Dean speaks in some detail of how citric, vitamin C, lemon and vinegar interact with heroin of varying purity. Six interview participants mention using lemon juice for injection preparation. Dean is the only one who continues to use household acids on a frequent basis:

[I use lemon and vinegar] loads of times, maybe for every ten times I inject probably two or three times, yeah, because I use so much citric for one bag, like the whole sachet, I run out of it constantly.

His use of lemon juice also appears excessive: “it stings quite a lot because generally I don’t put water in with it, I just use neat lemon juice … I’m destroying my, now you know why my veins are so knackered.” While attributing venous damage to lemon juice use he was unaware of its potential to cause candida infection *(*“No, I haven’t heard about that. How does it affect your eyes?”*)*. Other participants were aware of the risks, with some using other household materials to mitigate damage where no alternative to lemon was possible:

[In prison] if you’re going to use lemon juice … get a teabag and pour it through a teabag so you’re making it as pure as you can, getting all the little bits out. … See all the little bits of pith and that, when you cook up and draw it up, that’s what going to make you go blind and shit like that, and it’s quite scary. (Mason)

#### Constraint and convention: ‘one for one’

While ‘experts’ generally took ownership of acidifier overuse, drawing on the logics of experiential knowledge and preference, a distinct group of participants attributed their injecting practice to external factors. Here, excess use of acidifier was accounted for in relation to structural or information constraints or as following convention set by sachet size. For those referencing constraint, excess use was recognised as such and, where possible, injecting practice changed in response. Those referencing convention seldom illustrated movement in practice, using “1 for 1” [one sachet for one bag] was something they had ‘always done’ and was rarely subject to reflection. The following exchange is indicative:

M.H: How much citric would you put in for say a £10 bag?

Tim: Well they come in little sachets, so one of them.

A whole sachet?

Yeah.

Have you always used a whole sachet?

Yeah …

Do you use a whole sachet just because that’s how much there is in the sachet or because that’s how much you feel you need to get it to work properly, to get it to dissolve?

No, that’s how much is in the thing, just rip it open, yeah.

While ‘experts’ might add an acidifier gradually, increasing the amount until the mixture was clear (even with awareness that this was not needed), those responding to convention would generally use a whole sachet at once; the act of ‘opening it up and tipping it in’ requiring little thought or modification. Isaac explains: “I put it all in at once because I always been like that, I put gear in then I put the citric in, it just does it automatically, does that make sense?” For these participants, expertise is located with injection equipment manufacturers and providers—sachet size is an assumed guide to good practice. As Mason says: “most people think a sachet’s for a bag.”

Participants referencing structural or information constraints might also speak of using ‘1 for 1’ but in the past tense—in relation to past uninformed or constrained practice. This was often with some bitterness and regret:

I used to use the full packet, I used to think you had to use the full bleedin’ packet, I mean I didn’t know, no one likely to say oh hang on, you didn’t use, you don’t need to use all that. (Alex)

Ray similarly speaks of learning to inject in relative isolation from peer-based knowledge or expert guidance:

We didn’t take care or know how to inject properly, so our veins were fucked quick, really quick. [MH: why were they fucked?] Oh, not rotating, probably using too much citric and using the wrong sized needles.

Both participants explicitly relate their early use of ‘1 for 1’ to venous damage, with practice modified over time in response:

When I started getting like citric burns and that, I used to put a little bit less in and see what it would do, yeah, because you don’t need to use all that much do you. (Alex)

Ray drew on manufacturing and access constraints to account for his early practice, with an associated plea for transparency of process, clear information and availability of alternatives:

Oh I was whacking a whole bag in, yeah, because that’s another thing as well I mean I’m sure the machine that puts it into the sachets, it can either, that’s the least amount they can set it to, or if they put it any less the amount of moisture that seeps into the packet it just, you know, it’ll ruin it, but I don’t know, it should be more clear on the packet, or they should … In London, when you go to the chemist they only give you citric, they won’t give you vit C.

For others, constraints operated both at the level of equipment provided and power dynamics inherent to their injection practice. Kirsty did not know how to prepare heroin and crack for injection and relied on her partner to both prepare and administer each injection:

MH: Do you see your man cook up? Do you know how much citric he puts in and stuff?

Kirsty: A whole bag. … it really burns. Really burns

He might be using a bit too much citric.

Yeah. That’s what I told him. “No it’s not, no it’s not”. [He gave me] a mouthful of abuse ... And he knows I need it, so what can I say? I’ve just got to accept it haven’t I?

Whether Kirsty’s partner would be open to modifying his practice in relation to ‘expert guidance’ (for example, information printed on each sachet packet) is unknown. However, the witnessed dynamics of his relationship with Kirsty made it clear that her requests would be more likely to entrench rather than shift potentially harmful drug preparation practice, particularly if he took on an ‘expert’ role within the relationship.

Given the constraints and convention attributed to acid sachet size, all participants were asked which and how much acidifier they used before sachet availability. Most spoke of buying citric or vitamin C in bulk from pharmacies or grocery stores and using only ‘a pinch’ during drug preparation. As Marie relates: “I bought it from the chemist, big tub of that, only cost about £1 or £2 and it lasts you forever … you put a tiny sprinkling in”. Katy expressed a continued preference for pharmacy sourced vitamin C powder, and like others, the quantity sourced appeared to positively impact amount used: “You just use little bit, little bit, because it’s a big tub … just little pinches”. When asked if this was likely to be less than from a sachet, she replied “Yeah, yeah, yeah, I think you do, I think you do [use less]”*.* Preference was also subject to constraint, with some no longer able to source vitamin C powder: “they used to sell them in little orange tubs in Boots but now they’re just tablets and I don’t know, because they’re orange tasting tablets I don’t really want to.” (Matt).

#### Attributing pain: normalisation and modification of practice

Painful injections, with a burning sensation experienced on administration, were common to participant accounts. For some, painful injections resulted in a modification to practice—with less or a different acidifier used. For others, this pain appeared to be normalised—an accepted part of the injection process, with little or no injection preparation modification. All attributed painful injections to the use of acidifier—primarily citric acid. Jade provides a visceral rendition of ‘citric burn’:

Citric, it burns your skin and you can feel it through your veins, and it’s a horrible feeling … When it burns it brings up all the veins, if you miss a hit oh my God, the citric tears you down, I’m talking about it can take you to the point where you’re whole hand turns red you know, all your hand, the middle of your palm is red because of the citric*.*

Many articulated a causal pathway from acidifier use to painful injections and venous damage: “you have the hit and then that vein’s destroyed because of the citric” (Mason). Some modified their practice in response:

I put as little [citric] as possible. I actually put less than other people, “oh put the whole bag in” and no, I put like a pinch in and I was aware of what it was and I thought this stuff is painful so I’m, like, little as possible … I really do think personally that it’s one of the major causes of losing your veins. It’s not the drug, it’s the Vit C or the citric. (Ian)

While Ian speaks of vitamin C and citric in the same context, most participants made a clear distinction. For some, citric was preferred due to its perceived ‘strength’ while those concerned with pain and venous damage tended to favour vitamin C:

Vit C is wicked because when I was on like, finger veins and shit, if I used citric that’s it, one use, that vein is gone. If I use vit C I could get a week out of one. (Ray)

Preference for citric tended to align with a normalisation and acceptance of painful injections, with little associated modification to drug preparation practice. Dean notes, “I’ve had loads of citric burns, yeah, it stings like fuck…. [but I] still do it, still use the whole sachet now”. Ian normalised injection site skin reactions and pain, attributing them to “the citric … normally you get that when you get a vein, it’s just part and parcel, isn’t it?” Here, modification was more likely to take place during drug administration than preparation—with potentially risky consequences:

What happens is like I’ll suddenly get this citric burn and then I’m forced to go looking elsewhere and then I have to go deeper and I have to use the 2ml barrel pins to get deeper. (Matt)

This movement from use of peripheral to deep veins, such as the jugular or femoral, is associated with increased risk of complications and SSTI [28]. Tim comments that now he uses deeper, bigger veins he no longer feels the citric burn as he did in his peripheral veins. Emma’s modified practice is also associated with additional risk: “I put too much citric in and it started burning me you know, so I had to take it out, it was too much, I … skin popped it.” Purposeful arterial injecting was also described, with pain negotiated through administration practice:

You’ve got the little digits [on the syringe], if you put a ten unit in or more the burning will be too much to take. What you do is you put a little, like, two bit, one of the little lines in, you wait, it’ll burn, it’ll die down, with another bit it’ll burn, it’ll die down and you’re like that and that’s how you do it in your artery. (Logan)

Participants less likely to reduce their acidifier use (‘experts’ and those following convention) were more likely to normalise pain. Those who spoke of a modified practice acted to avoid pain, and critiqued perceived structural constraints to doing so. While above, Matt attributes an almost malicious intent to the manufacturers of acids for injection: “it’s like they want you to burn yourself or something”, Jade states that current provision lacks insight into the needs of PWID:

I think that they should find other things that’s not going to damage people’s skin and all that. The citric burns your skin, I don’t think it’s fair … it’s bad enough people have a habit, but at least help them. I mean it is great, I’m grateful that they do, they gave us needles at the time, but when these people that pack these bags, they’re not people that use. If they was a user they would have more of an insight of what a person would be happy with … citric would be out of bounds … instead of citric, vitamin C is the best.

Matt references the constraints of sachet design while providing another narrative of pain, venous damage and loss:

Like a couple of times I’ve opened the packets and they’ve been quite full and they’ve just exploded over the spoon and just gone everywhere onto it and it ended up being like more than what I would normally put on. And yeah, my skin sort of just bubbles up and my veins just disappear immediately if I use too much citric … it’s like a burn, literally like a burn … it travels from where the veins split off … it will go quite a way, yeah, good foot and a half I’d say.

## Discussion

Injecting practice is shaped by a complex interplay of social and environmental factors [29] and is not uniformly productive of risk. Perceptions of and importance afforded ‘risk’ among PWID are also variable, informed by access to resources, hierarchies of priority, temporal orientation, environmental constraints, agency to effect change, and social relations and norms [30]. Qualitative accounts highlight the situated rationalities at play in injection preparation practice, with attributions of expertise mapping onto preparation practice and its variation or stability over time.

In combination with quantitative results, we posit that excessive acidifier use in injection preparation is common among PWID in the UK and can play a causative role in venous damage and associated sequelae. The proportion of participants reporting excessive acidifier use align across datasets, with 36% of the survey and 37% of the interview participants reporting typical use of more than half a sachet of acid in injection preparation with nearly a third (30% and 31%, respectively) using a whole sachet or more.

A limitation of the quantitative data is that variation in acidifier use over time is not ascertainable; the question pertaining to acidifier quantity lacks time period specificity. Given we employ the present tense to ask participants how much acidifier they typically *would* use when preparing a £10 bag of heroin, it is likely that answers reflect recent practice. Qualitative data provide a more nuanced picture of use over time and supports interpretation of quantitative data as pertaining to current use. Interview accounts illustrate 37% *currently or recently* using excessive acidifier and that for the majority, the amount used remained relatively stable throughout their drug use trajectory. This was particularly the case for participants who located their acidifier use within an expert-based practice (whereby the quantity used is determined by visual cue of injection clarity) and for those who located their practice in relation to external expertise (with quantity used determined by packet size). Some illustrated change over time, with acidifier use generally decreasing in relation to information gained or pain on injection practice. For these participants, constraint rather than expertise informed early acidifier use, with critical reflection provided on both the limitations of manufacturing guidelines and information provision. Data triangulation indicates that quantitative results likely pertain to current practice, with typical amounts of acidifier used across the injecting career higher for a sub-sample. This has implications for analyses pertaining to health harms, given variable duration of progression from venous damage to SSTI and associated complications.

Quantitative analyses show no significant associations between quantity of acidifier use and SSTI. Qualitative accounts, however, paint a vivid picture of ‘citric burn’ and proximal venous loss. Pain on injection was common, and unanimously attributed to the use of acidifier—particularly citric acid. For some, this led to the use of deeper veins for injection, including the femoral vein. Qualitative analyses helped inform questions asked of statistical analysis, including in relation to indications of venous damage such as femoral injecting. We found statistically significant associations between excessive acidifier use (> ½ sachet), femoral injecting and DVT. This, in combination with reference to the literature and qualitative analyses, supports our hypothesis of a causal pathway between acidifier overuse and venous damage. For example, Harris and Rhodes [13] detail the suffering and frustration experienced by PWID who have difficulty accessing damaged peripheral veins. Half of their qualitative London sample transitioned to using the femoral vein, even though it was positioned as ‘a last resort’ by many. Our survey sample also comprises a high proportion (42%) of PWID reporting femoral vein injecting, indicating peripheral venous damage.

Our data support earlier studies demonstrating that, for many PWID, the end point of injection preparation is determined by a clear solution, when all drug material appears from visual inspection to be dissolved [2, 31]. This is problematic given components in the injection such as adulterants, excess bicarbonate in crack cocaine, and plant-based materials in heroin may not necessarily dissolve at the same pH as the drugs themselves. The excess acid required to dissolve these materials increases injection solution acidity but not psychoactive drug content. ‘Expert’ participants demonstrated awareness of this but were reluctant to take the leap of faith required to use less acid in a context of uncertain drug quality. Here, rather than educational interventions, the optimum point of intervention becomes the drug itself—or the acid used to prepare it. Participants who externalised expertise rarely problematized their acidifier use, with trust placed in the convention of sachet size as a guide to good practice. Given variation in practice, with ‘experts’ adding acidifier step-wise dependent on visual cue of drug clarity and those adhering to convention habitually using a whole packet at once, intervention at the level of equipment manufacture is also desirable. For participants referencing constraint, some who initially may have adhered to convention, clear information provision was required. Below, we consider each of these intervention points: the drug, the acid, equipment manufacture and information provision, exploring constraints and enablers to each in the UK policy environment.

### The drug

While there are many variables impacting venous damage, femoral injecting and SSTI risk, it is crucial to understand how heroin source, form, chemistry and quality informs local drug preparation and administration practice in order to intervene at the level of health harm. Geographical variation in heroin source and form has implications for SSTI prevalence. In the US, for example, cities with a dominance of Mexican-sourced “Black Tar” heroin (BTH) report 40% higher rates of SSTI compared with cities supplied by Colombian sourced powder heroin [16, 32]. London participants using Afghanistan sourced brown base heroin report painful injections and vein damage, unlike Philadelphia participants using salt form heroin [8]. In a context of prohibition, heroin quality is variable and difficult to determine. This also has implications for injection preparation practice. Our data illustrate that even with awareness of the ‘ideal amount’ of acid to use, many PWID will use more to dissolve poorly soluble cutting agents or adulterants such as paracetamol and quinine.

Structural problems require structural solutions. Diamorphine hydrochloride, pharmaceutical grade heroin, is readily soluble in water with a resultant pH of 4, which is less likely to cause vein damage. Department for Health registered doctors can legally prescribe pharmacetical grade heroin in the UK, but this is not supported by policy in practice. As beginning to be recognised with ‘party drugs’, such as MDMA, adulterants can cause significant health harms. Pill testing has gained traction and not before time [33]. In the absence of quality control, testing for the diamorphine content of heroin and confirmation of its presence in the base form would allow calculation of the amount of acid needed in preparation [2]. The logistics, feasibility and acceptability of this approach are, however, unclear.

### The acid

Given current legislative constraints to the provision of safer drugs for injection or testing to determine quality, modifications to acidifier provision require consideration. NSP throughout the UK primarily provide citric acid sachets, and vitamin C is less readily available. The question of which acid represents a lesser risk of harm has been subject to laboratory experiment [9]. Laboratory testing of injections prepared using methods replicated from PWID found citric acid produced injections with a lower pH (which will be more locally irritating) and vitamin C produces injections with a higher osmolality (could cause vein damage if given rapidly) [9]. As larger quantities of vitamin C are needed to convert base drug to a soluble form, there is a greater margin of error; excess vitamin C use will not be as irritating as excess citric acid. High amounts of vitamin C in injection preparation can cause precipitation (a solid clouding in the injection solution); a potential deterrent for excess use. There is a case to be made, therefore, for phasing out citric acid distribution in favour of vitamin C. In response to presentation of our data [34], the pan-Dorset Harm Reduction group, in consultation with service users, has removed citric acid from Dorset NSPs in favour of vitamin C [35].

Ciccarone and Harris [8] found that the pH of heroin solution prepared with vitamin C (3.4) was still too acidic for healthy veins. They propose development and supply of another mild, yet effective organic acid. The provision of sterile buffered solutions (acidic solutions in which the extent of the pH drop is controlled by the addition of harmless chemicals) could be an alternative to vials of water for injection plus acid sachet. Such buffering is routinely used in the manufacture of pharmaceutical injections, and a range of chemicals are approved for use by medicines licencing agencies. The challenge lies in regulation, with such products likely to be classified as medicinal products. Manufacturers are then subject to onerous medicine regulation, testing and licencing. For individual PWID, a pinch of sodium bicarbonate (baking soda) could be added at the end-stage of drug preparation to buffer the solution. This practice is incorporated into injection preparation by some PWID in New Zealand, where citric and other acidifiers are used in the preparation of heroin from morphine sulphate tablets, ‘homebake’ and opium [36]**.**

### Equipment manufacture and provision

Modifications to the manufacture and supply of acids currently available for injection may be the most feasible option in the short term. Some participants expressed a preference for vitamin C but found it difficult to access. ‘Choice’ of acidifier is constrained by availability – injecting supply bags provided through pharmacy NSP often only contain citric acid. Providing sachets of citric and vitamin C in each pack or promoting choice in other ways could support uptake of vitamin C. Sachet size will, however, still pose a constraint to good practice. This is a potent signifier of appropriate quantity, and use of one sachet for one bag of heroin (‘1 for 1’) was common among our participants and in the few other studies where details of acidifier use are documented [3, 9, 10]. As a ‘single use’ item, one sachet is generally provided for each needle and syringe. Information to use less than a whole sachet is present on a small proportion of sachets from one manufacturer only. Options for acting on the constraint of sachet size include: diluting acid strength with a harmless bulking agent; revisiting the use of pharmaceutical grade manufacture to provide smaller ‘food-grade’ sachets; adding a scoop in or alongside sachets; returning to bulk provision. The content of vitamin C sachets could be reduced from the current 300 mg weight to a lesser weight that theoretically provides enough acid to dissolve a ‘typical’ street deal of heroin, such as 150 mg. All of these options contain their own constraints, none are ideal.

**Fig. 1:**
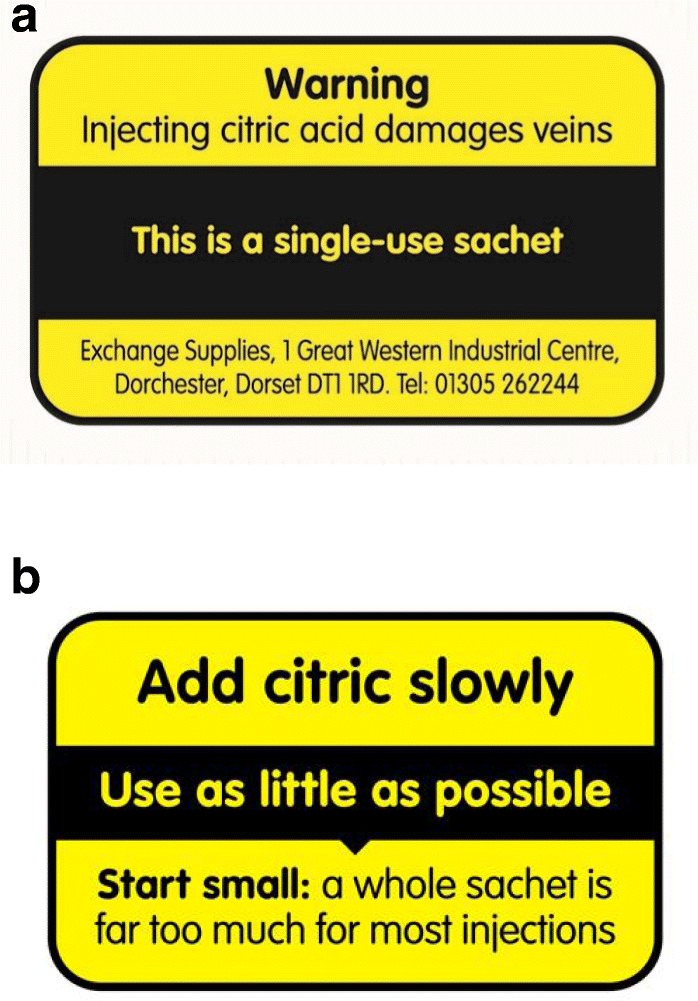
**a** Exchange Supplies Citric Acid Sachet, Old design. **b** Exchange Supplies Citric Acid Sachet, New design (October 2019)

The dilution of citric acid with a harmless soluble powder approved for injection manufacture before the 100 mg fill weight is measured could offer some protection when a ‘1 for 1’ approach is taken, especially for PWID who adopt this as routine practice. It would not obviate against the use of additional acidifier to dissolve cutting agent residue. Acidifier sachets as currently supplied are not considered medicinal products by the regulator (MHRA). It is less clear whether this would be the case if dilution of the acid was undertaken prior to fill. Reducing the sachet size of citric acid is not possible if adhering to industry Good Manufacturing Practice sachet filling standards (where 100 mg is the lower limit) but could be possible if supplied to ‘food grade’ standard which is not subject to weight standardisation constraints. Single use sachets of acidifier were developed to support optimal injecting practice, in which injections are prepared for and by the individual with no equipment shared. The contents are sterilised to reassure providers that products supplied to PWID support pharmaceutical injection preparation much as can realistically be achieved. Some participants, however, recalled using smaller amounts of acid for preparation when sourced from larger pots. The presence of a quantity in the pot obviously in excess of requirements paradoxically seems to have promoted stepwise addition of smaller amounts. Removal of sterilisation constraints and/or return to bulk provision could meet resistance due to concerns about risk of bacterial infection from the acid itself. Given these constraints, and in response to our findings [34], one UK manufactor has collaborated with us to change the messages on their citric and vitamin C sachets to stress that “a whole sachet is far too much for most injections” (see Fig. 1).

### Reinvigorating harm reduction messaging

‘Expert’ accounts drew on practical and theoretical knowledge to both justify and undermine the excessive use of acidifier—primarily in relation to the preparation of snowballs and poor quality heroin. The distinction between preference in practice and ‘logical’ knowledge is problematised in a context of uncertainty. When “you try to dissolve it *because you don’t know what it is*” (Mason), both logic and preference are undermined. To leave residue in the spoon requires trust—a ‘leap of faith’—that injection strength will not be compromised. Familiarity with and trust in the science of heroin preparation can aid such a leap. A harm reduction video, demonstrating the chemistry of street heroin preparation, provides an innovative response to such need [37]. Here, a heroin injection is prepared in a laboratory setting, with small amounts of citric acid added in a stepwise process and the chemistry involved explained. Available in DVD and through online social media, the video targets PWID and drug service practitioners, but participant accounts suggest limited awareness with no evidence of uptake by drug treatment services.

There is an evident need for clear information to support heroin preparation practice, particularly given PWID uncertainty about acidifier use reported in multiple UK studies [8, 11]. Retrenchment of social and public health services in the UK coupled with a ‘recovery’-focused drug policy agenda has decimated drug treatment services and the capacity and confidence of staff to provide fundamental safe injecting advice. The provision of non-stigmatising services attuned to the priorities of PWID, such as vein care and injection preparation, can help to reengage marginalised PWID and prevent health harms [13]. Safe injecting sessions, including information on drug preparation chemistry, should be integral to service provision including pharmacy-based needle and syringe provision. Clear and consistent messaging is required on all acid sachets, with pamphlets detailing optimal injecting practice and preparation in all equipment packs. While important, these initiatives must not act in isolation from structural change. An individualisation of responsibility, common to health promotion messaging, can be counterproductive in the context of constraint—acting to further stigmatise and marginalise rather than empower. There is little use, for example, informing of the need to use sterile equipment or to favour vitamin C if equipment access is constrained.

## Conclusion

Venous damage and chronic injecting-related problems are common to the experience of PWID. To reduce injecting-related injury and associated consequences, we contend that it is crucial to understand the interplay of environmental and practice-based risks underpinning venous damage among PWID. The use of acid in injection preparation can precipitate vein damage and is amenable to structural and practice-based change. In the absence of policy reform to enable provision of safer drugs for injection, there is an urgent need to revisit injecting equipment design and distribution in order to alleviate health harms and distress among the most marginalised. This could include modification to the form and/or packaging of acids currently available. This paper evidences research impact on practice, with modifications made to acidifer supply (Dorset) and sachet design (Exchange Supplies) in response to findings dissemination. These are welcome first steps, incorporating educational intervention with structural change.

## Data Availability

The datasets used and/or analysed during the current study are available from the corresponding author on reasonable request.

## Footnotes

1 Sachet: a small sealed packet with an airtight seal and waterproof membrane. See: http://bit.ly/citricinfo
